# Anticancer activity of metformin: a systematic review of the literature

**DOI:** 10.2144/fsoa-2019-0053

**Published:** 2019-08-22

**Authors:** Mohamad Aljofan, Dieter Riethmacher

**Affiliations:** 1 Department of Biomedical Sciences, School of Medicine, Nazarbayev University, Nur-Sultan 010000, Kazakhstan

**Keywords:** AMPK, anticancer, cancer, mechanism of action, metformin

## Abstract

**Background:**

The anticancer activity of metformin has been confirmed against several cancer types *in vitro* and *in vivo*. However, the underlying mechanisms of metformin in the treatment of cancer are not fully understood. This systematic review aims to discuss the possible anticancer mechanism of action of metformin.

**Method:**

A search through different databases was conducted, including Medline and EMBASE.

**Results:**

A total of 96 articles were identified of which 56 were removed for duplication and 24 were excluded after reviewing the title and abstract. A total of 12 research articles were included that describe different antiproliferative mechanisms that may contribute to the antineoplastic effects of metformin.

**Conclusion:**

This analysis discussed the potential anticancer activity of metformin and highlighted the importance of AMPK as a potential target for anticancer therapy.

## Lay abstract

Metformin is a widely used antidiabetic drug. It is the recommended treatment for Type II diabetes. Recently published reports claimed that the antidiabetic drug can also protect patients against cancer. This has led to a widespread interest in the antidiabetic medication as a possible treatment for cancer. While studies have confirmed the anticancer potential of metformin, the way in which metformin improves cancer outcome remains unknown. Hence, the current study investigates the reported mechanisms that explain how the antidiabetic drug works as an anticancer agent.

Type II diabetes mellitus is a metabolic disorder that leads to cardiovascular complications. What is more, people with diabetes have an increased risk of several common cancers [1–4] as well as a higher mortality rate than the normal population [5]. Metformin is an oral biguanide agent that was the US FDA approved in 1994 and is the recommended first-line treatment for Type II diabetes mellitus [6,7]. It is believed that metformin improves glycemia by acting on the liver via AMPK activation [8].

Metformin is known to reduce hepatic gluconeogenesis and increase skeletal muscle glucose uptake by activating AMPK, which is a cellular energy sensing enzyme that regulates cellular energy status by undergoing phosphorylation and increasing activity when ATP levels decrease and AMP levels increase. The change in the ATP:AMP ratio is used as an indicative marker of energy deficiency [9].

There is a continual increase in the prescription and usage of metformin; data from the Quality Outcomes Framework in England show that across the whole of the last decade, prescribing of metformin has more than doubled, from 9.4 million items in 2006/2007 to 20.8 million items in 2016/2017 [10]. Prescribing for Type II diabetes mellitus has changed considerably, with metformin rising to account for 91.0% of first-line therapy among newly diagnosed patients with T2DM and 79.9% of add-on therapy for patients on sulfonylureas [11].

In addition to its on-label use as an antidiabetic medication, metformin has other off-label uses, such as for the treatment of polycystic ovary syndrome, hyperinsulinemia, prediabetes, obesity and metabolic syndrome. Data from the National Disease and Therapeutic Index (NDTI) database showed that the most common diagnoses with metformin use were diabetes (34·9%), followed by metabolic syndrome (20·9%), polycystic ovary syndrome (17.2%) and obesity (6.5%) [12].

Interestingly, the antidiabetic drug is receiving extensive attention as a potential anticancer treatment following retrospective reports that showed improved survival rate in different cancer types for diabetic patients using metformin. The drug is gaining international interest for its potential use to treat/prevent different types of cancer, cardiovascular disease, ageing and neurological disorders [13]. Data from *in vitro* and preclinical studies confirmed the anticancer activity of metformin against several types of cancer, which promoted the initiation of more than 55 clinical trials that aimed to investigate the potential anticancer effect of metformin against endometrial, prostate, pancreas, lung and breast cancer [14].

However, the underlying mechanism of action of how metformin exerts its anticancer activity is still not completely understood. Thus, the current manuscript is a systematic review of the literature that aims to analyze and characterize the different reported mechanisms of the anticancer activity of metformin.

## Methods

The current review was performed according to the guidelines shown in the PRISMA statement for reporting systematic reviews and meta-analyses of studies. That evaluate health care interventions: explanation and elaboration by Liberati *et al.* [15].

### Search strategy

Articles published within the last 15 years to 15 February 2019 were searched through the available databases including; Medline via PubMed and EMBASE via Elsevier. We used ‘Metformin’, ‘metformin mechanism of action’ and ‘metformin and cancer’ as the search terms. The search was restricted to English language studies including journal articles, theses and conference proceedings.

### Study selection & quality

M Aljofan and D Riethmacher carried out the study search, study selection, as well as quality assessment and any disagreement was solved by discussion. The quality of the studies included were assessed according to the quality of the body of evidence The GRADE approach [16].

### Types of study selected

Experimental studies including *in vitro* and/or *in vivo*.

#### Types of outcome

The primary measures of interest include reported mechanism of actions of metformin other than its hypoglycemic activity. Secondary measure was the anticancer effect of metformin.

#### Data extraction

The extracted data included the name of the first author; publication year; methodology and findings.

#### Publication bias & limitations

In this review, we aimed to analyze the reported mechanisms of antitumor action of metformin, thus, we only included studies with *in vitro* analyses, which do not necessarily reflect all aspects of the organism as a whole. Some studies were not included in the review for reasons including subject duplication, article language and no/limited access to articles. Another limitation is that some of the reported mechanisms have not yet been reconfirmed. This may be in part due to the fact that this topic, anticancer activity of metformin, is relatively new and that most of these studies were published within the last decade or so.

## Results

**Figure 1.**
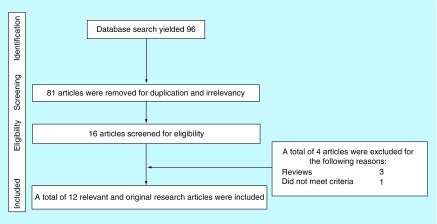
Flow of the information searched through the current systematic review. The different stages of search for the current systematic review. The initial search yielded 96 articles based on their titles, but the majority were excluded after reviewing the abstract. The remainder were screened and only 12 articles were included in the review.

The database search produced 96 articles and no other articles were identified using other sources. A total of 56 articles were removed for duplication and 24 articles were excluded after reviewing the title and abstract. Full text of the 16 remaining articles was obtained and reviewed for the final inclusion. Three review articles and one irrelevant study were further excluded. Thus, a total of 12 studies were included in the current review. Figure 1 shows the flow of information that was searched through the different phases of the current systematic review. Table 1 shows the list of articles included in the review and a brief summary of the findings.

**Table 1.** List of studies included in the review.

No	Study	Title	Year	Study design	Outcome	Ref.
1	Zakikhani *et al*.	Metformin is an AMP kinase-dependent growth inhibitor for breast cancer cells	2006	Used insulin sensitive breast cancer cells	Metformin acts as a growth inhibitor rather than an insulin sensitizer for epithelial cells	[17]
2	Deng *et al*.	Metformin targets Stat3 to inhibit cell growth and induce apoptosis in triple-negative breast cancers	2012	The effect of metformin was tested in four different human breast cancer cell lines	Metformin inhibits Stat3 activation (P-Stat3) at Tyr705 and Ser727 and downstream signaling	[18]
3	Liu *et al*.	Activation of AMPK by metformin promotes renal cancer cell proliferation under glucose deprivation through its interaction with PKM2	2019	The action and mechanism of metformin were confirmed in two renal cancer cell lines and BALB/C nude mice	Antiproliferative effects of metformin in cancer cells are highly dependent on the glucose concentration in the extracellular environment	[19]
4	Ma *et al*.	Low glucose and metformin-induced apoptosis of human ovarian cancer cells is connected to ASK1 via mitochondrial and endoplasmic reticulum stress-associated pathways	2019	The activity of metformin was confirmed using three ovarian cancer cell lines and in mouse xenograft model	Metformin induces ER stress and cell apoptosis, and ASK1 plays an important role in the antitumor effect of metformin *in vivo*	[20]
5	Hart *et al*.	SPHK1 is a novel target of metformin in ovarian cancer	2019	The activity was confirmed using human serum samples, ovarian cancer cell lines and nude mice model	Anticancer activity of metformin may be via the regulation of SPHK1 and S1P expression	[21]
6	Wang *et al*.	Metformin induces human oesophageal carcinoma cell pyroptosis by targeting the miR-497/PELP1 axis	2019	The mechanism of action was determined using human oesophageal carcinoma cells and immunodeficient mice	Mechanistically, metformin induces pyroptosis of ESCC by targeting miR-497/PELP1 axis	[22]
7	Lu *et al*.	Metformin triggers the intrinsic apoptotic response in human AGS gastric adenocarcinoma cells by activating AMPK and suppressing mTOR/AKT signaling	2019	The study was performed *in vitro* using human AGS gastric adenocarcinoma cell line	Metformin induces apoptosis may involve ERK, JNK and p38 MAPK-regulated pathways in AGS cells	[23]
8	Tseng *et al*.	Metformin treatment suppresses melanoma cell growth and motility through modulation of microRNA expression	2019	The mechanism of action was determined in two different human melanoma cell lines	Metformin treatment suppressed the motility and growth of melanoma cells due to direct modulation of miR-192-5p-EFEMP1 and miR-584-3p-SCAMP3 axes in melanoma cells	[24]
9	Hanawa *et al*.	Antitumor effects of metformin via indirect inhibition of protein phosphatase 2A in patients with endometrial cancer	2018	Human data followed by confirmation of mechanism of action using two endometrial cancer cell lines HEC265 and HEC1B	Metformin reduced the expression of PP2A in patients with EC and that the reduction of PP2A expression was related to the antiproliferative effect of metformin	[25]
10	Wu *et al*.	An Ancient, unified mechanism for metformin growth inhibition in *Caenorhabditis elegans* and cancer	2016	The results were obtained using Hela Cells and *C. elegans*	Anticancer activity of metformin is attributable to its inhibition of mitochondrial complex I	[26]
11	Zordoky *et al*.	The antiproliferative effect of metformin in triple-negative MDA-MB-231 breast cancer cells is highly dependent on glucose concentration: implications for cancer therapy and prevention	2014	The results were obtained using triple-negative MDA-MB-231 breast cancer cells	The antiproliferative effect of metformin thought to be achieved through an AMPK-dependent mechanism	[27]
12	Chen *et al*.	Metformin suppresses cancer initiation and progression in genetic mouse models of pancreatic cancer	2017	This is an *in vivo* study using genetically modified mice model (oncogenic Kras-mediated PDAC) mouse models	The anti-angiogenic effects of metformin is confirmed and that the mechanism is likely through AMPK and or STAT3	[28]

EC: Endometrial cancer; ER: Endoplasmic reticulum; ESCC: Esophageal squamous cell carcinoma; PDAC: Pancreatic ductal adenocarcinoma.

### Confirmation of anti-angiogenic activity of metformin

Mounting evidence from *in vitro*, *in vivo* and epidemiological and observational studies reported that metformin may be an effective treatment or adjuvant for cancer therapy. Initially, in an observational study, Evans *et al.* reported a link between the use of metformin and a reduced risk of cancers and cancer-related deaths [29]. The study reported that metformin use may reduce the risk of cancer in patients with Type II diabetes in a dose–response relationship. Several studies followed.

For instance, *in vitro* studies that have reported the putative anticancer effect of metformin including Wu *et al*., which reported the effect using iterative genomic screening in *C. elegans*, identified a genetic pathway linking metformin to the inhibition of cancer growth and lifespan extension [26]. Also, Zordoky and colleagues investigated the antiproliferative effect of metformin using triple-negative MDA-MB-231 breast cancer cells. The results indicated that metformin can significantly inhibit MDA-MB-231 cell growth [27].

Results from animal studies such as that of Chen *et al*. in 2018, which studied the effect of metformin in genetic mouse models of pancreatic cancer, claimed that metformin can inhibit tumor growth. They treated the mice with a daily dose of metformin starting at 6 weeks of age. They showed that mice treated with metformin presented a prolonged overall survival, and decreased tumor volume and tumor weight [28]. What is more, the study found that intake of metformin delayed pancreatic tumorigenesis in a cancer mouse model, suppressed chronic pancreatitis-induced tumorigenesis and showed a promising effect in reducing chronic pancreatitis-induced pancreatic desmoplastic reaction.

Hanawa *et al*. presented a study using human data that examined the anti-angiogenic effects of metformin on patients with endometrioid carcinoma, which is considered the most common histologic type of endometrial carcinoma and of uterine malignancy overall [30,31]. The study included 27 patients who received metformin for 4–6 weeks prior to the day of scheduled surgery. Initially, they received 750 mg/day; this increased weekly up to 1500 or 2250 mg/day. The results reported that preoperative metformin treatment significantly reduced the expression of protein phosphatase 2A (PP2A-B), which is considered a hallmark of antiproliferative effects of metformin administration [25].

### LBK-dependent AMPK activation

In 2006, a study by Zakikhani *et al*. aimed to investigate the molecular antineoplastic mechanism of metformin on epithelial cells [17]. The authors hypothesized that metformin exhibits direct antiproliferative actions on epithelial cells. They examined the efficacy of metformin on human breast cancer cells (MCF-7), which is known to be sensitive to insulin and IGF [32].

However, instead of enhancing insulin or IGF-I-stimulated growth, metformin acted as a growth inhibitor, an action that the authors claimed to be associated with a suppression of p70S6K phosphorylation. Furthermore, they examined the effect of metformin on cellular proliferation using several cancer cell lines including the LKB1 null HeLa cell lines. Except, for LKB1 null HeLa, dose–response treatment on several cell lines resulted in growth inhibition, suggesting that the antiproliferative activity of metformin is most likely achieved via LKB1 signaling, which aligns with growth inhibition seen on the untransformed MCF-10A human breast epithelial cells [32,33].

Furthermore, the authors investigated whether the metabolic actions of metformin require AMPK activity similar to that in other tissues such as muscles or hepatocytes. They not only found that metformin stimulates AMPK phosphorylation, but also that the phosphorylation was achieved in a dose-dependent manner. To confirm these findings and to determine whether activation of AMPK by metformin is required for the antiproliferative effects of metformin, the authors used siRNA against AMPK α1 subunit. Interestingly, the AMPKα1 siRNA reduced the stimulatory effect of metformin on AMPK phosphorylation and that the reduction was correlated with the levels of reduced AMPKα1.

This study suggests that metformin’s activation of the AMPK pathway is not confined to hepatocytes but can be observed in epithelial cells as well. In conclusion, the authors claimed that metformin’s antiproliferative activity was achieved via an LBK-dependent pathway.

### Inhibition of STAT3 activation

Deng *et al*. studied the antiproliferative antisurvival activity of metformin against six triple-negative breast cancer cell lines, and found that metformin induced growth inhibition on all of these cell lines [18].

The study showed that metformin significantly reduced both tyrosine and serine phosphorylation of Stat3 (P-Stat3 at Tyr705 or Ser727), reduced P-mTOR and induced P-AMPK/AMPK. Also, the study showed that metformin inhibits Stat3 activation, either directly or indirectly, through a time- and dose-dependent manner.

To further explore the role of Stat3 in metformin action, in one experiment they transfected these cell lines with constitutively active Stat3, and in another experiment knocked down Stat3 expression using a lentiviral system.

Interestingly, constitutively active Stat3 expression reduced metformin-associated changes in cell growth and apoptosis as well as cell signaling in all of the tested triple-negative cancer cells. Whereas, specific knockdown of Stat3 expression enabled metformin to significantly induce more growth inhibition in each of the Stat3-knockdown cells compared with the control.

In order to investigate the association between the anticancer activity of metformin and mTOR, the authors compared the growth inhibition and apoptotic effects of metformin alone or combined with the selective Stat3 inhibitor S3I-201 to that of the mTOR inhibitor rapamycin. The study found that both metformin and S3I-201 alone induced apoptosis, while the combination was even more potent; however, rapamycin treatment alone induced mild growth inhibition, but failed to significantly enhance the metformin-associated growth inhibition when combined with metformin, which indicates that metformin’s effect was not due to its effect upon mTOR.

The study concluded that the anticancer activity of metformin is achieved via the direct or indirect activation of Stat3. Unlike Zakikhani *et al.*, the study claims that the metformin activation of Stat3 and hence, its antitumor activities are independent of mTOR.

### Metformin antitumor activity is glucose dependent: high glucose improves metformin activity

Since cancer cells encounter nutrient limitations, a study by Liu *et al*. investigated the effect of nutritional environment on the anticancer activity of metformin using renal cancer cells. The authors examined whether poor outcome of metformin in renal cancer cells may be influenced by the nutrient condition [19]. In this study, the authors used a model of glucose deprivation to study the effect of nutrient limitation in renal cancer cells under metformin treatment.

The study indicated that cellular treatment with metformin under normal condition resulted in a significant suppression of cell growth, but that changing the cellular environment from normal to glucose-deprived reversed the metformin-induced growth suppression and that metformin appears to promote cell growth under this condition.

This finding was aligned with that of Zordoky *et al*., which investigated the antiproliferative effect of metformin using triple-negative MDA-MB-231 breast cancer cells. The study suggested that treatment with metformin did not inhibit the growth of MDA-MB-231 cells cultured in hyperglycemic conditions. However, MDA-MB-231 growth was inhibited by metformin when normoglycemic conditions were used for cell culture.

Unlike previous reports, Liu and colleagues suggested that metformin activation of AMPK is not affected by the environment, and that treatment with metformin did not produce obvious apoptosis in renal cancer cells under either normal or glucose-deprived conditions. However, several reports have found the exact opposite and that low glucose environment was reported to increase AMPK activation [2,18,34–36].

To further test the effect of nutritional environment/food starvation on anticancer activity of metformin, the authors injected 1 × 10^6^ A498 cells into nude mice, which was supposed to mimic an *in vivo* form of renal cancer, and restricted their dietary intake to resemble the effect of glucose deprivation. Food starvation alone slowed, but did not suppress, tumor growth. However, the study suggested that under glucose starvation, metformin treatment did not suppress the tumor growth, but in fact increased its volume.

In addition, the authors tested the expression of proliferation marker, Ki-67, and reported that metformin treatment suppressed the expression of Ki-67 under normal conditions, but induced its expression under glucose deprivation. Additionally, the *in vivo* analysis of Ki-67 revealed that metformin treatment promoted cellular proliferation in food-starved nude mice and that metformin treatment may increase renal cancer cell proliferation under glucose deprivation. In conclusion, they suggested that the antiproliferative effects of metformin in cancer cells are highly dependent on the glucose concentration in the extracellular environment.

### Metformin antitumor activity is glucose dependent: low glucose improves metformin activity

A study by Ma *et al*. investigated the anticancer mechanism of action of metformin on different human ovarian cancer cells [20]. The study claims that metformin treatment in low glucose environment enhances ovarian cancer cell cytotoxicity by apoptosis induction via mitochondrial pathway, which was confirmed by the increased ratio of Bax/Bcl-2.

Also, the combination of low glucose and metformin was shown to enhance the expression of cytosolic cytochrome c and reduce the mitochondrial membrane potential in cancer cells compared with normal glucose with or without metformin, indicating that cell apoptosis is triggered by combination of low glucose and metformin, via the mitochondria-associated pathway. These findings suggested that mitochondrial dysregulation plays an important role in apoptosis that was induced by the combination of low glucose and metformin.

Additionally, metformin and low glucose treatments resulted in a significant increase in the expression of Noxa, which is a pro-apoptotic protein associated with mitochondrial damage, and a substantial decrease in the expression of the anti-apoptotic protein, Bcl-2. Intriguingly, the treatment combination of low glucose and metformin induced the phosphorylation of the ASK1-dependent pathway, which is a key regulatory component of Noxa expression [21,37,38].

To determine the involvement of ASK1 in the subcellular localization of Noxa protein, cells were pretreated with NQDI-1, a pharmacological inhibitor of ASK1. The results showed that the total amount of Noxa and the level translocated to the mitochondria were significantly decreased, and the expression of Bcl-2 was increased, suggesting that ASK1 plays an essential role in low glucose and metformin-induced subcellular localization of Noxa.

To further confirm the role of ASK1, knockdown of ASK1 expression using siRNA reduced the loss of mitochondrion membrane potential and apoptotic ratio in ovarian cancer cells treated with the combination of low glucose and metformin, which suggests that low glucose and metformin-induced mitochondrial damage are the consequence of ASK1/Noxa pathway activation.

Furthermore, the study shows that ASK1 activation is associated with accumulation of reactive oxygen species (ROS) and further activates downstream signaling including JNK. The analysis of the role of ROS in the activation of ASK1 and JNK in low glucose environment and metformin treatment demonstrated that the inhibition of ROS decreased the phosphorylation level of ASK1 in cells treated with the combination of low glucose and metformin. These results suggest that the accumulation of ROS may have been involved in the low glucose and metformin-induced loss of mitochondrion membrane potential.

Also, the pretreatment of cancer cells with the ROS scavenger NAC reduced several effects seen with the combination of low glucose and metformin, including a significant decrease in the loss of mitochondrion membrane potential, a decrease in the mitochondrial localization of Noxa and a significant inhibition of caspase 3 activity in cancer cells, suggesting that ROS accumulation is associated with Noxa-mediated mitochondrial damage.

Of note, the study suggested that the combination of low glucose and metformin activate endoplasmic reticulum stress through ROS that triggers endoplasmic reticulum (ER) stress-associated apoptosis through ROS/ASK1/JNK pathway. Thus, the study confirmed the antitumor effect of metformin in ovarian cancer cells *in vivo*, and showed that metformin decreased tumor volume and weight in a dose-dependent manner as well as effectively induced ASK1 phosphorylation.

Finally, they claimed that the treatment with metformin alone effectively increased the expression of Grp78, GADD153 and cleaved caspase 3, suggesting that metformin treatment in the xenograft model could induce ER stress and cell apoptosis. In conclusion, the study suggested that metformin induces ER stress and cell apoptosis, and showed that ASK1 plays an important role in the antitumor effect of metformin *in vivo*.

### Metformin causes apoptosis via inhibition of S1P

A recent study by Hart *et al*. investigated the effect of metformin on sphingosine kinases. These bioactive lipids are a singular group that is believed to regulate tumor progression [38], including the apoptosis- and cell-inducing ceramides and sphingosine, as well as S1P, which promotes cell growth, proliferation and migration [21,39,40]. Cancer cell fate is influenced by the balance of ceramide/sphingosine and S1P – this is referred to as the sphingolipid rheostat [21,40].

First, the authors wanted to determine whether S1P and SPHK1 promote ovarian cancer, cancer cell migration and invasion. The results of wound closure assays demonstrated that exogenous S1P promoted migration of ovarian cancer cells and that this effect was attenuated by pretreatment with metformin. Also, they confirmed that S1P promoted invasion of the ovarian cancer cell line and that metformin treatment reduced this effect.

Second, direct evaluation in ovarian cancer cell lines (in which endogenous levels of SPHK1 are nearly undetectable) of the effect of SPHK1 overexpression on several hallmarks of cancer growth, showed that cells with ectopic SPHK1 expression had a higher rate of migration and proliferated more rapidly than control transfected cells, confirming that SPHK1 could indeed promote tumorigenicity.

To determine whether metformin modulates the S1P rheostat in ovarian cancer, analysis of serum metabolites from patients with stage III/IV ovarian cancer was performed. This demonstrated that ovarian cancer patients who were using metformin for the treatment of Type II diabetes mellitus had significantly lower serum S1P levels than patients not using metformin.

These results were further supported in an *in vitro* experiment that aimed to investigate the effects of metformin on the sphingolipid rheostat, where an ovarian cancer cell line was treated with metformin or control. The results confirmed that metformin-treated cells had markedly reduced S1P and sphingosine levels while ceramide levels were increased. Based on these findings the authors claimed that metformin shifts the rheostat toward reduced S1P production.

### Metformin induces apoptosis via ERK, JNK and p38 AMPK-regulated pathways & mitochondrial ROS

Lu and colleagues used human gastric cancer AGS cells to investigate the antiproliferative effect of metformin on cancer cells and potentially determine the underlying apoptotic mechanisms [23]. After treating the cells with different drug concentrations over several time points, the study suggested that metformin suppressed cancer cell growth via the induction of apoptosis in a concentration and time dependent manner. The study also indicated that metformin at 20, 30 and 40 mM was able in a concentration-dependent manner to produce double-stranded DNA fragmentation, which is a unique biochemical hallmark of apoptosis. The results of caspase-3/7 activity analysis indicated that metformin (20, 30 and 40 mM) significantly enhanced the activity of caspase-3/7 in a concentration-dependent manner, which demonstrates the ability of metformin to trigger apoptosis of AGS cells that may be caspase-3/7-dependent.

In order to investigate the molecular pathway involved in the anticancer activity of metformin, and whether or not AMPK is involved, AGS cells were treated with an AMPK inhibitor, compound C and then p-AMPK (indicative of AMPK activation) and cell viability were analyzed.

The results demonstrated that compound C suppressed phosphorylation of AMPK and significantly reversed the effect of metformin on cell viability compared with metformin treatment only. This suggests that, for AGS cells, modulated AMPK signaling mediates metformin-induced apoptosis. Further analyses of the phosphorylation of AKT (p-AKT), mTOR (p-mTOR) and p70S6K (p-p70S6K) demonstrated that metformin decreased the phosphorylation of AKT, mTOR and p70S6K without affecting protein expression, which indicates that metformin enhances apoptosis potentially by targeting AMPK and AKT/mTOR pathway in AGS cells.

Based on different analyses the authors claimed several potential theories of how metformin induces apoptosis. For instance, the study claimed that the apoptotic mechanism of metformin may involve ERK, JNK and p38 MAPK-regulated pathways in AGS cells, or through an increase in mitochondrion ROS, or through an intrinsic signaling that induces mitochondria-mediated caspase-dependent apoptosis.

### Metformin targets miR-497-PELP1 to induce pyroptosis

The recently published study by Wang *et al*. investigated the possible antitumor mechanism of metformin on human esophageal cells [22]. The study aimed to decipher the role of PELP1 in the progression of esophageal squamous cell carcinoma (ESCC). They studied the role of PELP1 systematically using the Oncomine database and *in vitro* by measuring the mRNA and protein levels of PELP1 in different ESCC cell lines. The data from both of these tests confirmed that not only the DNA copy, but also the mRNA and protein levels of PELP1 are increased in human ESCC.

The study suggested that PELP1 plays an important role in ESCC recurrence and elevated levels of PELP1 in ESCC lead to poor prognosis. This led the researchers to investigate the role of metformin on PELP1 using two ESCC cell lines that were treated with different concentrations of metformin over different time periods. The results indicated that metformin downregulated the levels of PELP1 protein and mRNA in a dose- and time-dependent manner.

The study claimed that cellular treatment with metformin upregulates miR-497, which is an miRNA that was shown to be significantly downregulated in cancer tissues [41]. Interestingly, the study suggested that PELP1 is a target for miR-497 and that upregulation of miR-497 will in turn downregulate PELP1. Furthermore, a daily intraperitoneal injection of metformin at 250 mg/kg bodyweight for 4 weeks in ESCC animal model resulted in a significant reduction in the size and weight of the tumor.

What is more, RT-qPCR results indicated that the mRNA levels of PELP1 and miR-497 were down- and upregulated by metformin, respectively. Further analyses showed that the levels of PELP1 in tumors carried by metformin-treated mice were significantly reduced and the levels of GSDMD, which is a characteristic of pyroptosis, were significantly increased. GSDMD interacts with membrane phospholipids to form pores in the plasma membrane that eventually leads to pyroptosis, which is a nontraditional programed cell death characterized by pore-formation on the plasma membrane resulting in cell swelling and plasma membrane disruption.

Based on both the *in vitro* and *in vivo* findings, the study claimed that metformin exerts its anticancer activity by the induction of nontraditional programed cell death in ESCC through targeting the miR-497-PELP1 axis.

### Metformin modulates miR-192-5p-EFEMP1 & miR-584-3p-SCAMP3

Like Wang *et al*., the recent study by Tseng and colleagues also supports the notion that metformin is likely to exert its anticancer activity via miRNA regulation [24]. The study used next-generation sequencing (NGS) to perform small RNA profiling to identify metformin-regulating miRNAs and explore the effects of miRNAs on antimelanoma cell growth and motility.

The results showed a significant reduction on the migration ability and motility of human melanoma cell lines A2058 and A375 after a 3-day treatment with 5 mM of metformin. Also, flow cytometry analysis of melanoma cell cycle after metformin treatment indicated that the number of cells at the S and G2/M phases increased and the number of cells at the G0/G1 phase decreased compared with those in the control group. Likewise, a significant increase in the population of apoptotic cells after 5 mM metformin treatment was noticed. These results indicated that melanoma cell growth could be suppressed after metformin treatment through impairing cell cycle progression and inducing cellular apoptosis.

Analyses from the next gene sequencing indicated that metformin may produce different gene expression in different cell lines. For example, metformin treatment resulted in an increase in 41 types of miRNAs expression and a reduction of 35 types of miRNAs expression in A2058 cells, but the results were different in A375 cells where the treatment resulted in a significant upregulation of 27 types of miRNAs and downregulated 28 types of miRNAs.

However, based on the fact that metformin suppressed the growth of melanoma cells, it should have upregulated tumor-suppressive miRNAs and downregulated oncogenic miRNAs. Thus, out of the many upregulated/downregulated miRNAs, the study selected miR-192-5p and miR584-3p, which were previously verified to suppress tumors in human cancers to decipher metformin anticancer activity.

Interestingly, an overexpression of miR-192-5p and miR584-3p on melanoma cell growth was observed that resulted in a clear suppression of colony formation and invasion abilities as well as proliferation, which were partly improved after miR-192-5p and miR584-3p inhibitor transfection.

Furthermore, microarray analyses identified several potential target genes for miR-192-5p and miR-584-3p including the two oncogenes *EFEMP1* and *SCAMP3*, which were significantly decreased after transfection with miR192-5p and miR-584-3p mimics, respectively.

They used siRNA to investigate the effects of *EFEMP1* and *SCAMP3* knockdown on melanoma cell growth. They found that *EFEMP1* and *SCAMP3* knockdown had considerably suppressed cell colony formation, proliferation and substantially induced cell cycle arrest at G2/M and increased the sub-G1 population. Furthermore, cell invasion and migration were clearly suppressed by *EFEMP1* and were not changed by *SCAMP3* knockdown. In conclusion, the results suggested that metformin treatment suppressed the motility and growth of melanoma cells due to direct modulation of miR-192-5p-EFEMP1 and miR-584-3p-SCAMP3 axes in melanoma cells.

## Discussion

Metformin (dimethylbiguanide) is the recommended first-line treatment for Type II Diabetes Mellitus [37]. Generally, it is assumed that metformin improves glycemia through AMPK activation and its effect on the liver [42]. However, growing evidence implies that metformin has other target organs including the gut and intestines [36,42].

In addition to its effect against hyperglycemia, the use of metformin was linked to possible reduction in risk of cancer and cancer-related mortality and that patients with diabetes using metformin were protected against different types of cancer, such as glioma, endometrial, breast, colon and gastric cancer [43–47].

**Figure 2.**
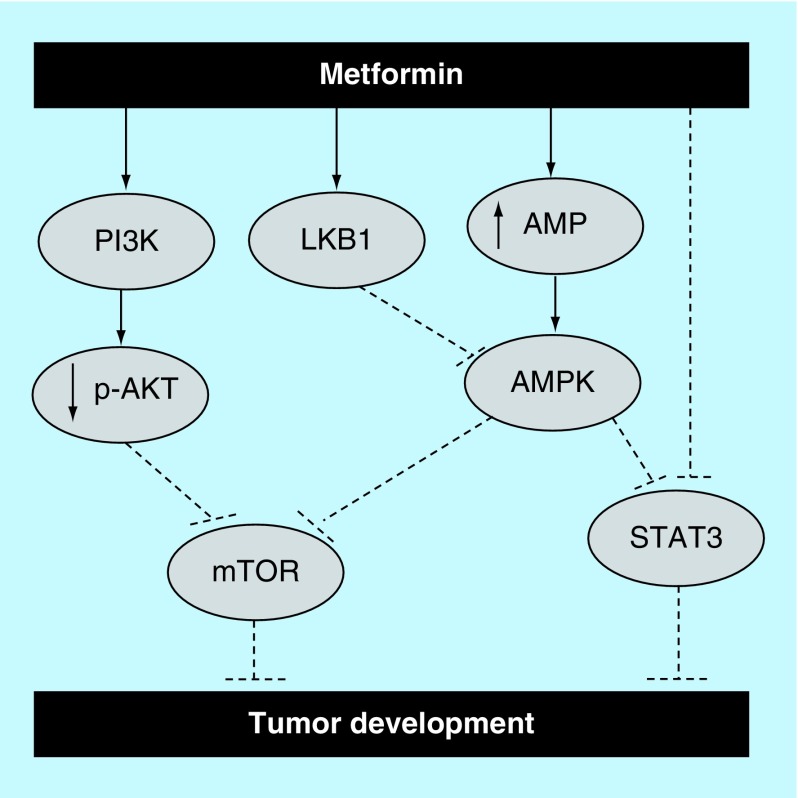
Potential antiproliferative mechanisms of metformin. Metformin’s potential activation of the apoptotic pathway. It is thought that metformin inhibits tumor growth and progression via activation of apoptosis. For instance, it was claimed that metformin enhances apoptosis by targeting AMPK and AKT/mTOR pathways. It was also reported that metformin activates Stat3 through an mTOR independent manner as well as via an AMPK/mTOR dependent way.

While the antitumor mechanisms of metformin are not yet known, several reports have confirmed the antiproliferative ability of metformin against different types of cancer through different mechanisms (Figure 2). For example, several *in vitro* studies demonstrated that treatment with metformin was capable of inhibiting cellular metastasis of EC109 esophageal squamous cell carcinoma cells [48], MG63 and U-2 OS osteosarcoma cells [49], and SiHa and HeLa cervical cancer cells [50].

Furthermore, in a dose- and time-dependent manner, metformin was shown to induce apoptosis in various cancer cell lines including SKOV3, A2780 and ES2 ovarian cancer cells [51], CAL 27, CAL 33 and UMSCC47 head and neck carcinoma cells [52], HepG2 hepatoma cells [53], B16F10 melanoma cells [54] and paclitaxel-resistant A2780-PR and cisplatin-resistant ACRP cells [51]. However, in this report we attempted to list and discuss the mechanisms that were reported as possible ways that metformin may induce its antitumor effects.

Metformin-stimulated activation of AMPK in cells other than hepatocytes will result in control of cellular proliferation [33], which is also supported by Zakikhan *et al*., who showed activation of the AMPK pathway by metformin is not confined to hepatocytes but can be observed in epithelial cells as well [17].

Furthermore, the result of AMPK activation in epithelial cells, such as in breast cancer tissue, may lead to reduced proliferation, general reduction of mRNA translation and protein synthesis. These findings are supported by several reports including a study by Stapleton *et al*. who suggested that in tissues other than liver and muscles, the α1 isoform, which was shown to be associated with cellular proliferation, is physiologically more important than the metabolism and energy demand associated α2 isoform [55]. Furthermore, a study investigating the effect of metformin on breast cancer indicated that metformin reduced cell growth by targeting the AMPK signaling pathway [23,56].

**Figure 3.**
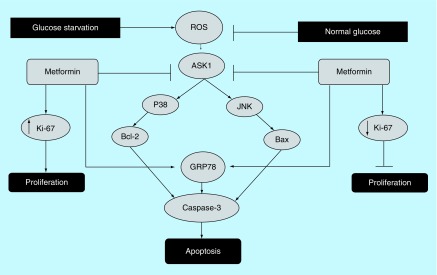
The effect of glucose environment on metformin’s antiproliferative activity. The effect of glucose levels of low/normal on the antiproliferative activity of metformin. Some reports have suggested that glucose starvation increases ROS production that triggers ER stress induced apoptosis through ROS/ASK1/JNK pathway or via Bcl-2 signaling, which is thought to enhance metformin’s antiproliferative activity. To the contrary, others reported that low glucose may hinder metformin’s antiproliferative activity and that treatment with metformin significantly reduced the expression level of the proliferation marker Ki-67 in normal glucose, but the opposite was observed with in glucose environment. ER: Endoplasmic reticulum; ROS: Reactive oxygen species.

On the contrary, it was reported that pretreatment with an AMPK antagonist inhibited prostate cancer cell proliferation [57,58]. Similarly, it was reported that the outcome of AMPK activation depends on the cellular environment such as that the antitumor effect of metformin-induced AMPK activation is highly dependent on the glucose concentration in the extracellular environment [59]. For instance, AMPK activation in a normal glucose environment will induce antiproliferative effects [34]. Also, an *in vivo* study showed metformin to have a reduced antitumor activity on control diet mice compared with high-energy diet mice associated with hyperinsulinemia and accelerated tumor growth [60]. Moreover, Liu *et al*. suggested that low glucose environment can directly influence the result of AMPK activation and that the activation of AMPK promotes renal cancer cell proliferation under stressful metabolic conditions (Figure 3). Similar findings suggested that AMPK activation mediated by lower ATP/AMP ratio, which represent a tumor microenvironment [35], promotes cellular survival under stressful metabolic conditions [61,62].

However, the observed survival in a low glucose environment may be due to the Warburg effect, where cancer cells reprogram their metabolism to promote growth, survival and proliferation [63]. AMPK activation is known to induce antiproliferative effects in many cancer cells, and it is known that glucose deprivation may temporarily inhibit this activity, but is very unlikely to be of a significant impact for several reasons. First, the Warburg effect was suggested in some instances to be a temporary effect [63]; second, prolonged glucose deprivation can induce an inflammatory response and increase the production of ROS, thus eventually damaging cell membrane and nucleic acids [64]. Arguably, a low glucose-induced raise in ROS will rather increase cellular apoptosis, thus contributing further to the antitumor activity of metformin, a theory that has been backed and supported by a number of studies including that of Menendez *et al*., which showed that the combination of metformin and glucose withdrawal were quite lethal to cancer cells [65].

In contrast, Ma *et al*., reported that glucose deprivation enhanced the antitumor effect of metformin, which aligns with earlier reports that showed the synergistic effect of combining low glucose with metformin-induced AMPK activation [65,66]. However, Ma and colleagues argued that the increase in antitumor effect of metformin does not depend on ROS, which they showed by cellular pretreatment with the ROS scavenger N-acetyl-l-cysteine.

However, a number of reports including that by Yang *et al*. proved that metformin activates ROS and induces ER-dependent apoptosis [67]. Furthermore, hypoglycemia [68], hypoxia [1], viral infections [69] and ER-Ca^2+^ [70] lead to an ER stress that causes a mitochondrial dysfunction and apoptosis [71], indicating that ROS play a major role in cancer cell apoptosis under glucose depletion environment and that prolonged ER stress activates apoptosis [72].

All of the studies included in the current report showed that metformin was able to, directly and indirectly, inhibit different cancer types *in vitro* and some have confirmed it *in vivo*. Metformin can directly act on cancer cells by targeting the AMPK pathway in tumor cells that control metabolism, angiogenesis, inflammation and cancer stem cells [73], or by inhibiting cancer growth and proliferation via reducing insulinemia and glycemia [74].

The studies reported a number of interesting potential mechanisms and external factors that could explain metformin’s antitumor effect. Of note, both the cancer cell sensitivity to metformin and the anticancer mechanism of actions reported were shown to be cell dependent, thus the different and sometimes conflicting mechanisms described might be due to the physiological differences between different cells. However, all of the reported theories of anticancer effect of metformin revolve around or are linked to AMPK activation and the external factors are mostly glucose related. Therefore, we can speculate that the antiglycemic activity of metformin represents an important means of its anticancer activity and that it is likely to exert its anticancer effect by targeting AMPK in cancer tissues.

## Conclusion

The current study is a systematic review of the literature that investigates metformin’s antiproliferative mechanisms. While the current findings provide an insight into the anticancer mechanisms of metformin, it also highlights the importance of AMPK as a potential target for anticancer therapy. In conclusion, the safety profile, the route of administration and the long history of use make metformin an ideal candidate for repurposing to include other uses as well. However, the significant efficacy of metformin to inhibit cancer growth proliferation *in vitro* and *in vivo*, as well as the large number of clinical trials that aim to further investigate its efficacy as a potential anticancer adjuvant or treatment, reflect the potential that this drug can offer and warrants the need to decipher the exact mechanisms of its anticancer activity.

## Future perspective

Metformin is a relatively safe drug that has been used as an antidiabetic medication for several decades, which makes it a good candidate for repurposing. Currently, the drug has several off-label uses including the treatment of symptoms of polycystic ovarian syndrome. While we believe that metformin will have new indications/uses, either off-label or in-label, added to the existing ones, the indications are unlikely to include anticancer as a monotherapy. Nonetheless, metformin may be more suited as an adjuvant or as a combination to anticancer regimen. Further studies are needed to identify cellular targets of metformin that could be utilized in anticancer treatments, as well as the role of its glucose lowering ability as a potential mechanism or contributing factor. Additionally, future research should focus on investigating metformin’s tissue distribution in normal and cancer cells, which will probably help in better understanding its anticancer mechanisms and improve its potential anticancer usage.

**Summary points**

- Metformin is a widely used antidiabetic drug that has been reported to have antiproliferative activity against several cancers.
- The antiproliferative mechanism of metformin remains unclear.
- This study investigates the reported mechanisms of action that may contribute to metformin’s antiproliferative activity.
